# Senescence: novel insight into *DLX3* mutations leading to enhanced bone formation in Tricho-Dento-Osseous syndrome

**DOI:** 10.1038/srep38680

**Published:** 2016-12-07

**Authors:** Na Zhao, Dong Han, Haochen Liu, Yue Li, Sing-Wai Wong, Zhengyi Cao, Jian Xu, Xiaowei Zhang, Tao Cai, Yixiang Wang, Hailan Feng

**Affiliations:** 1 Department of Prosthodontics, Peking University School and Hospital of Stomatology, Beijing, China; 2 Oral Biology Curriculum, School of Density, University of North Carolina at Chapel Hill, The United States; 3 Department of Biochemistry and Molecular Biology, Peking University Health Science Center, Beijing, China; 4 School of Physics and Electronic Information, Henan Polytechnic University, Jiaozuo, China; 5 Institute of Genomic Medicine, Wenzhou Medical University, Wenzhou, China; 6 National Institute of Dental and Craniofacial Research, NIH, Bethesda, MD, The United States; 7 Central Laboratory, Peking University School and Hospital of Stomatology, Beijing, China.

## Abstract

The homeodomain transcription factor distal-less homeobox 3 gene (*DLX3*) is required for hair, tooth and skeletal development. *DLX3* mutations have been found to be responsible for Tricho-Dento-Osseous (TDO) syndrome, characterized by kinky hair, thin-pitted enamel and increased bone density. Here we show that the *DLX3* mutation (c.533 A>G; Q178R) attenuates osteogenic potential and senescence of bone mesenchymal stem cells (BMSCs) isolated from a TDO patient, providing a molecular explanation for abnormal increased bone density. Both *DLX3* mutations (c.533 A>G and c.571_574delGGGG) delayed cellular senescence when they were introduced into pre-osteoblastic cells MC3T3-E1. Furthermore, the attenuated skeletal aging and bone loss in *DLX3* (Q178R) transgenic mice not only reconfirmed that *DLX3* mutation (Q178R) delayed cellular senescence, but also prevented aging-mediated bone loss. Taken together, these results indicate that *DLX3* mutations act as a loss of function in senescence. The delayed senescence of BMSCs leads to increased bone formation by compensating decreased osteogenic potentials with more generations and extended functional lifespan. Our findings in the rare human genetic disease unravel a novel mechanism of DLX3 involving the senescence regulation of bone formation.

Distal-less homeobox 3 (*DLX3*), mapped to 17q21.33, is a member of the distal-less vertebrate family (*DLX1-6*), which has a crucial function in the development and differentiation process^1^,^2^. Inactivated *Dlx3* in mice leads to transcription factor placental failure, suggesting *Dlx3* is indispensable in placental development^3^. *DLX3*, regarded as a homeodomain transcription factor in vertebrate, is required for hair follicle differentiation and cycling programs, for tooth morphogenesis, and for skeletal formation and development^1^,^4^,^5^,^6^. Mutations of *DLX3* are closely related to Tricho-Dento-Osseous syndrome (TDO; OMIM 190320), which is a disorder with autosomal dominant inheritance mainly characterized by kinky hair, thin-pitted enamel (with remarkable attrition), dentin hypoplasia, and taurodontism as well as increased thickness and density of cranial and mandibular bones. To date, only six mutations in the *DLX3* gene have been identified within minority family groups^7^,^8^,^9^,^10^ (Supplementary Fig. S1). Among them, the most common mutation in *DLX3* is a frameshift mutation (c.571_574delGGGG; G191RfsX66), resulting in recoding and truncating the C-terminal transactivating domain of the DLX3 protein^7^. However, the de novo missense mutation (c.533 A>G; Q178R), previously reported in our department, was found in the homeodomain of the encoded protein^11^.

The patients with the TDO syndrome, showing a marked increased bone mineral density (BMD) in endochondral and intramembranous bones, imply that *DLX3* exerts an essential role in bone formation. It has been identified that DLX3 is not only detected in osteoprogenitor cells, osteoblasts and osteocytes *in vitro*, but also is expressed in both developing and postnatal bones *in vivo*^12^,^13^,^14^. Studies have strongly suggested the importance of *Dlx3* in bone by directly regulating bone differentiation determinants, such as osteocalcin (*Ocn*), *Runx2* and osteoactivin^15^,^16^,^17^. Notably, the accrual of bone mass and density is observed in transgenic mice with *Dlx3* ablation in osteogenic lineage cells, suggesting that *Dlx3* has a negative effect on osteogenesis^14^.

Aging brings significant changes to skeletal system with gradually bone loss, as well as a shift in tissue microenviroment with cellular senescence in bone marrow, like osteoporosis^18^,^19^. The replicative bone marrow-derived mesenchymal stem cells (BMSCs) senescence encompasses a progressive loss of proliferation ability and a declining osteodifferentiation potential^20^,^21^. Misexpression of *Dlx3* in the basal proliferative layer of epidermis of transgenic mice induces cell cycle arrest and leads to premature terminal differentiation^22^. In normal human epidermal keratinocytes, exogenous DLX3 promotes cell cycle arrest by activating tumor suppressor gene p53 and cyclin-dependent kinase inhibitor p21, which play critical roles in cell cycle and senescence regulation^23^. Collectively, these data suggest a possible role for *DLX3* in senescence during epidermis proliferation and differentiation. However, whether *DLX3* function as a modulator of cellular senescence involving bone formation has not been experimentally investigated.

To address the effect of *DLX3* mutations on senescence during bone formation, we firstly isolated naïve BMSCs from a TDO patient with *DLX3* mutation (c.533 A>G; Q178R). Interestingly, an attenuated osteoblastic activity accompanying with a delayed cellular senescence was observed in the BMSCs harboring the c.533 A>G mutation when compared with gender- and age-matched healthy BMSCs. This finding was further verified and was mechanistically studied by transfecting plasmids of the wild type *DLX3*, the mutant *DLX3* (c.533 A>G) and the truncated *DLX3* (c.571_574delGGGG) into pre-osteoblastic MC3T3-E1 cells. Consistently, the attenuated bone loss and aging in the aged transgenic mice with *DLX3* mutation (c.533 A>G) suggest a regulatory role of *DLX3* in the context of senescence during bone formation.

## Results

### Isolation of BMSCs from the patient with TDO

The 27-yr-old female patient with typical TDO features was enrolled in this study (Supplementary Fig. S2A,B and C)^11^. Specially, thickened cortical and abnormally increased trabecular bone density of mandibular was clearly observed in the patient’s panoramic radiograph. The cephalometric radiograph of the patient showed a bossed frontal bone, a square jaw, increased bone density and thickness in parietal and calvaria bones, with a lack of pneumatization of the mastoids. Mandibular bone specimens of the patient were collected during implant surgery (Supplementary Fig. S3A). Bone marrow derived stem cells were isolated from the specimen (named as TDO-BMSCs) and reached 80% confluence after 14 days culture (Supplementary Fig. S3B). The missense mutation of *DLX3* (c.533 A>G; Q178R) was confirmed by Sanger sequencing in the blood sample as well as in the BMSCs of the patient (Supplementary Fig. S2D). Further, BMSCs isolated from three gender- and age-matched normal donors as controls (named as CON-BMSCs). The flow cytometry assay, which showed that both TDO- and CON-BMSCs were negative for the hematopoietic markers CD34 and CD45, and positive for the mesenchymal stem cell-associated markers CD73, CD90, and CD106, indicated that DLX3 mutation did not affect expression of stem cell markers (Supplementary Fig. S3C).

### TDO-BMSCs exhibit weaker osteogenic potential than CON-BMSCs

**Figure 1:**
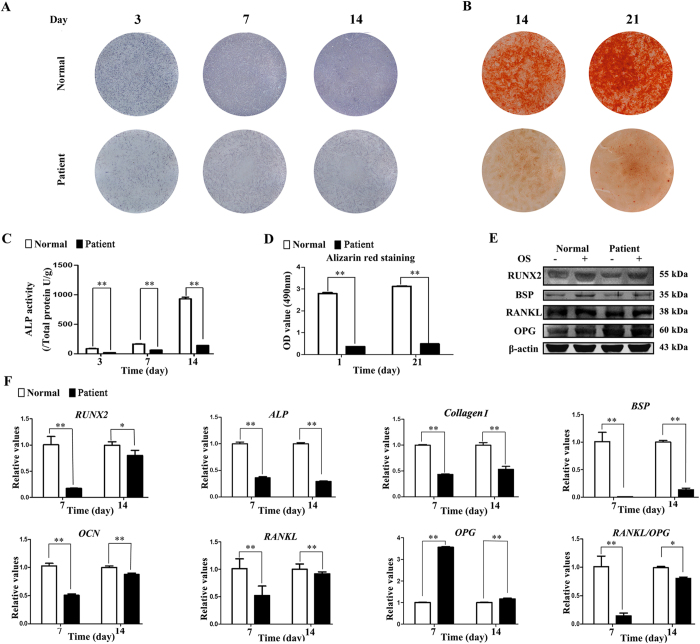
TDO-BMSCs exhibit weaker osteogenic potential than CON-BMSCs. TDO- and CON-BMSCs at passage 3 were cultured under osteoinduction medium. (**A**) ALP staining assay on days 3, 7, and 14 after osteoinduction. (**B**) Alizarin red staining assay at 14 and 21 days post-osteoinduction. (**C**) ALP activity assay on days 3, 7, and 14 after osteoinduction. (**D**) Quantification of alizarin red staining on days 14 and 21 post-osteoinduction. (**E**) Western blots of RUNX2, BSP, RANKL, OPG, and β-actin protein 14 days after osteogenic stimulation. (**F**) Osteogenesis-related genes (*RUNX2, ALP, COLLAGEN I, BSP, OCN, RANKL* and *OPG)* mRNA expression after osteoinduction. GAPDH served as an internal control. Data were presented as the mean ± S.D. of 3 independent experiments. *p < 0.05; **p < 0.01.

Due to the increased bone density in TDO patient, we firstly explored the inter-group differences on osteogenic potential. Under osteoinductive condition, the third passage of TDO-BMSCs differentiated into osteoblasts but showed lower levels of alkaline phosphatase (ALP) activity as assessed by ALP staining and ALP activity assays on day 3, 7, and 14 compared with CON-BMSCs (Fig. 1A,C). After osteoinduction for 14 and 21 days, Alizarin red staining (ARS) of calcium deposition with less staining intensity in TDO-BMSCs further confirmed these results (Fig. 1B,D). Western blot analysis indicated that the protein levels of RUNX2 and BSP were lower in TDO-BMSCs at 14 days post-osteoinduction (Fig. 1E). Meanwhile, by real-time PCR, the mRNA levels of *RUNX2, ALP, Collagen I, BSP*, and *OCN* were decreased in TDO-BMSCs on day 7 and 14 after osteoinduction, whereas the opposite effects were observed in CON-BMSCs (Fig. 1F). In addition, both real-time PCR and western blot analysis revealed lower levels of RANKL/OPG in TDO- relative to CON-BMSCs (Fig. 1E,F). Of note, DLX3 expression in TDO-BMSCs was half of that in normal controls (Supplementary Fig. S4).

### TDO-BMSCs exhibit slower senescence rate than CON-BMSCs

Since the panoramic radiograph indicated that the mandibular was heavily mineralized, decreased osteogenic potential apparently could not account for the phenotype of TDO. After literature reviewing, an important consideration is emphasized that bone formation is not only dependent on the osteogenic activity of individual osteoblast but also on the status of cellular senescence^18^,^19^,^24^. Of note, the classic senescence cell morphology, which was enlarged, flattened cellular morphology and a decline replicate proliferation, was observed in gender-, age- and passage-matched CON-BMSCs. Whereas, TDO-BMSCs, cultured for the same condition, showed relative younger state, with regularly long spindle-shaped fibroblast-like morphology (Supplementary Fig. S5). Thus, we examined whether DLX3 mutation intrinsically affected BMSCs senescence with further research.

**Figure 2:**
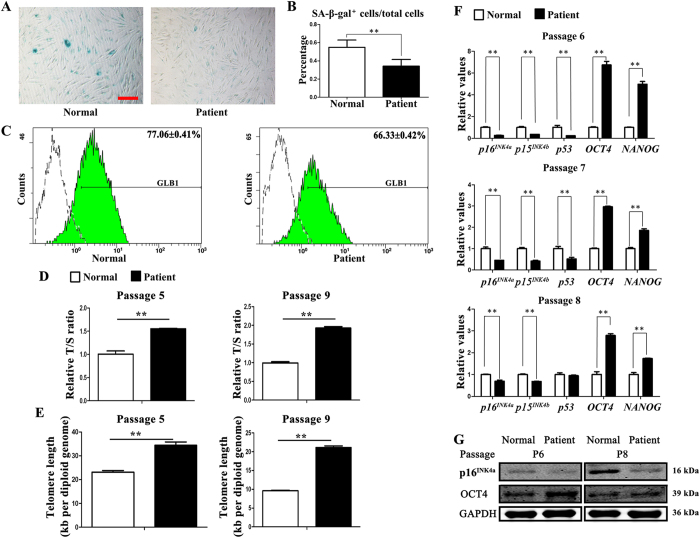
TDO-BMSCs exhibit slower senescence rate than CON-BMSCs. (**A**) Representative images of cells assayed for SA-β-gal activity (blue) (scale bar, 100 μm). (**B**) Percentages of SA-β-gal^+^ cells/total cells from experiments as in panel (A) β-gal activity normalized to total cells. (**C**) Flow cytometry of the percentage of GLB1 expression compared with isotype controls. (**D**) Relative telomere length expressed as relative T/S ratio measured by quantitative real-time PCR analysis showing remarkably longer telomeres of TDO-BMSCs at passage 5 and 9. (**E**) Absolute telomere length expressed kb/diploid genome determined by qPCR also displaying longer telomeres of TDO-BMSCs at passage 5 and 9. (**F**) Real-time PCR for *p16*^*INK4a*^, *p15*^*INK4b*^, *p53, OCT4*, and *NANOG* in TDO- and CON-BMSCs at the passages 6, 7, and 8. (**G**) Western blots of p16^INK4a^, OCT4, and NANOG protein levels; GAPDH served as an internal control. Data were presented as the mean ± S.D. of 3 independent experiments. *p < 0.05; **p < 0.01.

Senescence-associated β-galactosidase (SA-β-gal), defined as a β-galactosidase activity detectable at pH 6.0, has been used as a common marker for aged cells as it has been demonstrated to increase with aging of various cells both *in vitro* and *in vivo*^25^,^26^. The number of SA-β-gal^+^ staining cells 38% in TDO-BMSCs and 55% in CON-BMSCs suggested that BMSCs with DLX3 mutation remained a younger status while the normal BMSCs entered a premature senescence (Fig. 2A,B). Consistently, GLB1 expression, the gene encoding lysosomal β-galactosidase^27^, was measured by flow cytometry and showed a lower level in TDO-BMSCs (Fig. 2C).

Previous studies have identified that accelerated telomere shortening is observed in senescence cells^28^. Relative telomere length (expressed as relative ratio of telomere to single-copy gene (36B4) [relative T/S ratio]) and absolute telomere length (expressed kb/diploid genome) at passage 5 and 9 was measured by quantitative real-time PCR. It has been acknowledged that the decrease in the relative T/S ratio positively correlates with telomere shortening^29^,^30^. The results showed that the relative T/S ratio was higher in TDO-BMSCs (Fig. 2D). Moreover, absolute telomere length results further confirmed that TDO-BMSCs had longer telomeres (Fig. 2E).

p16^INK4a^, p15^INK4b^, and p21, encoded by *CDKN2A* gene, *CDKN2B* gene and *CDKN1A* respectively, together with tumor suppressive p53 transcription factor, were considered core elements of signaling pathways involved in cell senescence for their activation inducing cells into irreversible cell cycle arrest^31^,^32^,^33^. On the other hand, OCT4 and NANOG were stem cell specific transcription factors for maintaining pluripotency of stem cells during development^34^,^35^. The real-time PCR results showed that aging-related genes (*p16*^*INK4a*^, *p15*^*INK4b*^, and *p53*) were down-regulated and stem cell-associated genes (*OCT4* and *NANOG*) were up-regulated in the passages 6, 7, and 8 in TDO-BMSCs (Fig. 2F). Correspondingly, western blot analysis also exhibited that p16^INK4a^ protein expression was significantly reduced, while OCT4 expression was clearly increased in TDO-BMSCs (Fig. 2G). These results not only confirm that DLX3 mutation prevents the premature senescence of BMSCs but also indicates that DLX3 mutation stabilizes the pluripotency of BMSCs. Prolonged expression of stemness-related genes allow TDO-BMSCs to produce more “stem”-like osteogenic cells, to generate more daughter cells, and to extend the functional lifespan of osteoblastic cells to perform bone remodeling.

### Overexpression of WT-DLX3, MT-DLX3 and TR-DLX3 in MC3T3-E1 cells

To abrogate background difference generated by different donors’ cells and to further identify whether the novel mutation is a gain- or loss- of function, we constructed plasmids of wild type *DLX3*, mutant *DLX3* (c.533 A>G) and mutant *DLX3* (c.571_574delGGGG), and transfected into osteoprogenitor cells MC3T3-E1 to investigate their function on osteogenic differentiation and aging *in vitro*. Of note, the mutant *DLX3* (c.571_574delGGGG) was used to confirm the effect of DLX3 mutation on osteogenic potential and senescence.

**Figure 3:**
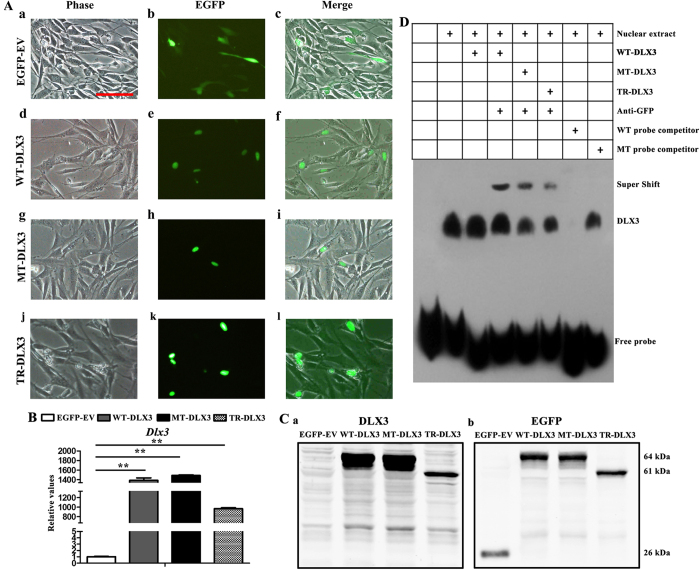
Cellular localization, DNA binding property to RUNX2 and overexpression of WT-DLX3, MT-DLX3 or TR-DLX3 in MC3T3-E1 cells. (**A**) Panels showing EGFP and EGFP-DLX3 fusion protein expression in MC3T3-E1 cells 48 h after transfection with pEGFP-C1, pWT-DLX3, pMT-DLX3 and pTR-DLX3. Differential interference contrast (DIC) images were acquired to visualize cell shape (a, d, g and j). Images of EGFP expression (b, e, h and k) were detected by fluorescent microscope. WT-DLX3, MT-DLX3 or TR-DLX3 was located in nuclear while the EGFP was in cytoplasm, which was shown in the merged images (c, f, i and l). (**B**) DLX3 mRNA expression was highly enhanced in WT-DLX3, MT-DLX3 and TR-DLX3 compared with EGFP-EV after 48 h transfection by real-time PCR. (**C**) DLX3-GFP protein expression was detected by western blot analysis with anti-DLX3 (a) and anti-EGFP (b) antibodies after 48 h transfection. Only EGFP protein was detected in EGFP-EV samples. EGFP-DLX3 fusion protein was detected in WT-DLX3, MT-DLX3 and TR-DLX3 samples. Data were presented as the mean ± S.D. of 3 independent experiments. **p < 0.01; Scale bars = 100 μm. (**D**) DLX3 occupies its affinity site in the RUNX2 promoter. EMSA assay was performed by using a biotin-labeled double-stranded probe (containing the DLX3 affinity site). Nuclear extracts (10 μg) from HEK 293 T cells expressing GFP-WT-DLX3, GFP-MT-DLX3 or GFP-TR-DLX3 were used. The wild type probe (GCATTTTGTAATTTATTTCAAAGC) and the mutant probe (GCATTTTGTGGGTTATTTCAAAGC) were used as unlabeled competitors at a 100-fold excess. The presence of specific complexes, including supershifted GFP/DLX3 in the complexes, are indicated.

The recombinant plasmids in empty vector pEGFP-C1, carrying wild type *DLX3* and mutant *DLX3* (c.533 A>G) and (c.571_574delGGGG) expression cassette were transfected into pre-osteoblastic cell line MC3T3-E1 and named as WT-DLX3, MT-DLX3 and TR-DLX3, respectively. MC3T3-E1 cells transfected with pEGFP-C1 empty vector (named as EGFP-EV) serves as control. The green fluorescence was detected under fluorescent microscope at 48 h post-transfection, and the results showed that transfection efficiency was over 30% (Supplementary Fig. S7). EGFP expression tagged to WT-DLX3, MT-DLX3 and TR-DLX3 was detected in nucleus, whereas EGFP expression in empty pEGFP-C1 was in cytoplasm, suggesting that whether *DLX3* missense mutation (c.533 A>G) or *DLX3* truncation mutation (c.571_574delGGGG) could enter into nucleus, like wild type *DLX3* (Fig. 3A). In addition, both real-time PCR analysis and western blot analysis showed that the expression of DLX3 was substantially increased in WT-DLX3, MT-DLX3 and TR-DLX3 cells (Fig. 3B,C).

Previous study have shown that *DLX3* acted as a molecular switch that regulated *RUNX2* expression throughout bone formation, we here tested the ability of mutant DLX3 to bind *RUNX2* promoter to determine whether deceased osteogenic potential associated with TDO-BMSCs were directly due to *DLX3* mutation. The electrophoretic mobility shift (EMSA) experiments (Fig. 3D) showed that endogenous DLX3 could bind to the specific oligonucleotide probe. WT-DLX3, MT-DLX3 and TR-DLX3 were also able to bind the probe. The DLX3 EMSA signal was specific, as determined by competition with a 100-fold excess of unlabeled probe (wild-type [WT] probe), but not by an unlabeled mutant probe (MT probe) bearing point mutations in the high-affinity site. In addition, the stronger band in the lanes with the transfected WT-DLX3, MT-DLX3 and TR-DLX3 could be supershifted with anti-GFP, confirming that WT-DLX3, MT-DLX3 and TR-DLX3 do specifically bind the high-affinity site probe.

### WT-DLX3 and TR-DLX3 increase while MT-DLX3 decreases cellular osteogenesis in MC3T3-E1 cells

**Figure 4:**
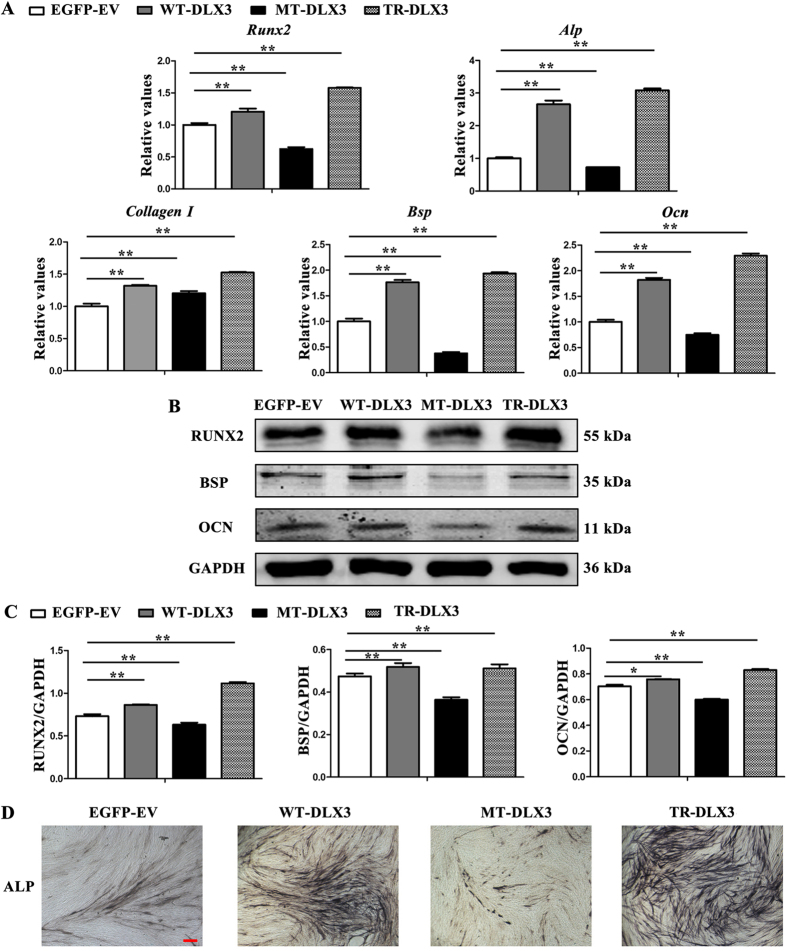
WT-DLX3 and TR-DLX3 increase while MT-DLX3 decreases cellular osteogenesis in MC3T3-E1 cells. MC3T3-E1 cells were transfected with pEGFP-C1, pWT-DLX3, pMT-DLX3 and pTR-DLX3 respectively. After 24 h transfection, these cells were further cultured in osteoinduction medium for 72 h. Then, the cells were harvested and subjected to real-time PCR and western blot. (**A**) Osteogenesis-related genes (*Runx2, Alp, Collagen I, Bsp*, and *Ocn*) mRNA expression were determined by real-time PCR. GAPDH served as an internal control. (**B**) Protein expression of RUNX2, BSP, OCN, and GAPDH were detected by western blot. GAPDH served as loading control. (**C**) Quantification of protein expression related to GAPDH. (**D**) ALP staining assay after osteoinduction for 72 h was performed. Data were presented as the mean ± S.D. of 3 independent experiments. *p < 0.05; **p < 0.01.

Since the mutations in DLX3 did not change the DNA bind activity to *RUNX2* promoter, we examined whether DLX3 mutations could affect osteogenic potential of MC3T3-E1 cells. After osteoinduction for 72 h, osteoblastic markers *Runx2, Alp, Bsp*, and *Ocn* mRNA expression detected by real-time PCR at mRNA level (Fig. 4A) and by western blot analysis at protein level (Fig. 4B,C) were slightly increased in WT-DLX3 and significantly increased in TR-DLX3, but remarkably decreased in MT-DLX3 when compared with EGFP-EV. ALP staining assay further confirmed these results by showing higher ALP activity in WT-DLX3 and TR-DLX3, lower in MT-DLX3 (Fig. 4D). These results suggest that different DLX3 mutations have different function on osteogenesis even though both of them could locate into nucleus and bind to *RUNX2* promoter. Of note, real-time PCR and western blot analysis showed that BMP2 expression was lower in TDO-BMSCs and was decreased in MT-DLX3 but increased in TR-DLX3 (Supplementary Fig. S8).

### WT-DLX3 pushes while MT-DLX3 and TR-DLX3 delays cellular senescence in MC3T3-E1 cells

**Figure 5:**
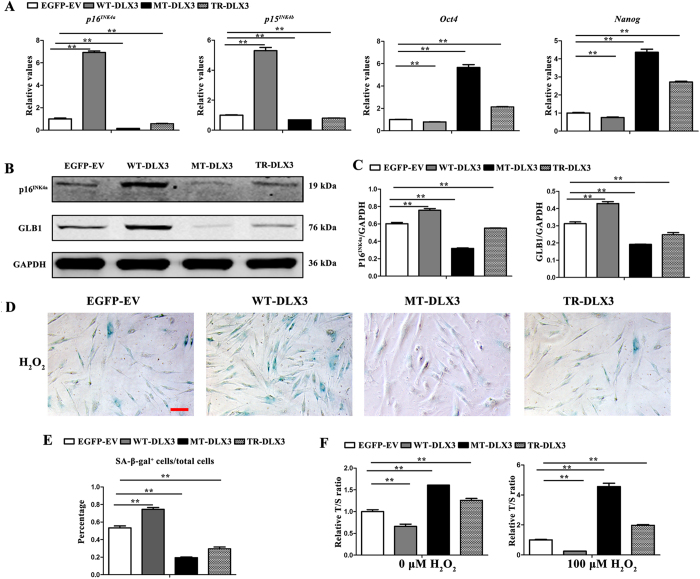
WT-DLX3 pushes while MT-DLX3 and TR-DLX3 delay cellular senescence in MC3T3-E1 cells. MC3T3-E1 cells were transfected with pEGFP-C1, pWT-DLX3, pMT-DLX3 and pTR-DLX3 respectively, and were further cultured for 72 h under osteodifferentiation induction. Then, the cells were harvested and subjected to do real-time PCR and western blot. (**A**) Senescence-related genes (*p16*^*INK4a*^ and *p15*^*INK4b*^) and stemness-related genes (*Oct4* and *Nanog*) mRNA expression were determined by real-time PCR. GAPDH served as an internal control. (**B**) p16^INK4a^, GLB1 and GAPDH protein expression were detected by western blot. GAPDH served as loading control. (**C**) Quantification of protein expression related to GAPDH. (**D**) SA-β-gal staining was performed in WT-DLX3, MT-DLX3 or TR-DLX3 overexpression-MC3T3-E1 cells exposed to 100 μM H_2_O_2_ for 96 h. Representative images of SA-β-gal-positive cells (blue) (scale bar, 100 μm). (**E**) Percentages of SA-β-gal^+^ cells/total cells from experiments as in panel (D) β-gal activity normalized to total cells. (**F**) Relative telomere length expressed as relative T/S ratio measured by quantitative real-time PCR analysis. Data were presented as the mean ± S.D. of 3 independent experiments. **p < 0.01.

Apparently, the inhibition effect of DLX3 mutation (Q178R) on osteogenic differentiation had been revalidated, we further examined its effect on senescence. After 72 h osteoinduction, aging-related markers *p16*^*INK4a*^ and *p15*^*INK4b*^ mRNA expression detected by real-time PCR (Fig. 5A) and p16^INK4a^ and GLB1 protein expression examined by western blot (Fig. 5B,C) were significantly increased in WT-DLX3 but decreased in MT-DLX3 and TR-DLX3 when compared with EGFP-EV. Stemness markers *Oct4* and *Nanog* mRNA expression detected by real-time PCR (Fig. 5A) were significantly decreased in WT-DLX3 but increased in MT-DLX3 and TR-DLX3 when compared with EGFP-EV.

Exogenous H_2_O_2_ has been used extensively as an inducer of oxidative stress *in vitro* aging models. Therefore, SA-β-gal staining and relative telomere length was performed in WT-DLX3, MT-DLX3 or TR-DLX3 overexpression-MC3T3-E1 cells exposed to 100 μM H_2_O_2_ for 96 h. The results showed that percentage of SA-β-gal-positive cells was significantly higher in WT-DLX3, but much lower in MT-DLX3 and TR-DLX3 (Fig. 5D,E). Telomere length assay by quantitative real-time PCR (Fig. 5F) demonstrated that T/S ration was aggravated in MT-DLX3 and TR-DLX3, but reduced in WT-DLX3, when compared with EGFP-EV. H_2_O_2_ exposure further confirmed the functional discrepancy between the *DLX3* mutations and wild type *DLX3*. These verified that both *DLX3* mutations had negative effect on senescence.

### DLX3 (Q178R) prevents skeletal aging and bone loss *in vivo*

**Figure 6:**
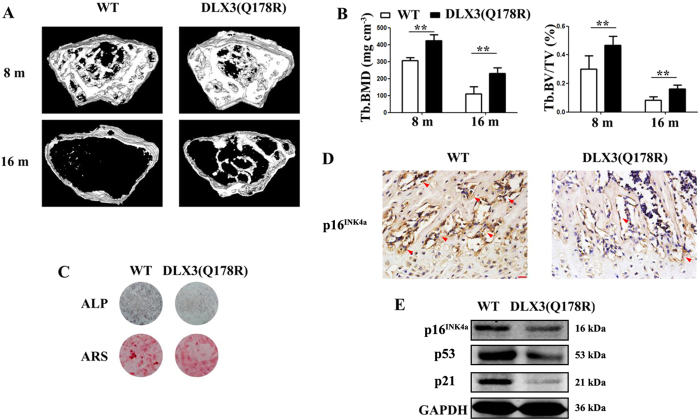
DLX3 (Q178R) attenuates skeletal aging and bone loss *in vivo*. (**A** and **B**) Micro-CT analysis: 3D trabecular reconstructions (**A**) Trabecular BMD (Tb. BMD), Trabecular bone volume ratio (Tb. BV/TV) (**B**) of distal femoral metaphysis regions from 8- and 16-months-old WT and DLX3 (Q178R)-Tg mice. (**C**) ALP staining and Alizarin red staining (ARS) of femur bone BMSCs from 8-months-old DLX3 (Q178R)-Tg vs WT mice, after osteogenic induction. (**D**) Immunostaining with p16^INK4a^ surrounding the trabecular bones from 8-months-old WT and DLX3 (Q178R)-Tg mice. Scale bars, 25 μm. (**E**) Western blot of p16^INK4a^, p53 and p21 in tibia and femur of 8 months mice.

Since DLX3 mutation (Q178R) could increase bone formation by preventing cell senescence even though with decreased osteogenic potential *in vitro*, we further generated transgenic mice expressing DLX3 mutation (Q178R) (named as DLX3 (Q178R)-Tg mice) to examine whether this novel mutation could protect against natural aging-mediated bone loss or osteopenia *in vivo*. Western blot analysis of DLX3 (Q178R) transgene was highly expressed in femur and tibia bone of founder mice (Supplementary Fig. S9A). Micro-CT analysis revealed that there was a gradual loss of trabecular bone mass with advancing age from 8 to 18 months in wild type mice (named as WT mice) (Fig. 6A). Further quantitative analysis showed that the most dramatic trabecular bone loss occurred between 8 and 16 months of age and average bone mineral density (BMD) dropped 62%, while bone volume/total volume (BV/TV) dropped 79%. However, the reduction was significantly less with DLX3 (Q178R)-Tg mice, with 43% in BMD and 65% in BV/TV respectively (Fig. 6B). Of note, we confirmed that *DLX3* transgene mRNA in bone tissues was still expressed in aged DLX3 (Q178R)-Tg mice using RT-PCR although the levels of DLX3 mRNA declined over age (Supplementary Fig. S9B). We also examined that whether natural aging-associated p16^INK4a^ activation was suppressed by DLX3 mutation (Q178R). While immunostaining of p16^INK4a^ expression revealed p16^INK4a^ activities in the vicinity of trabecular bones of WT mice. And p16^INK4a^ activities appeared less prevalent in DLX3 (Q178R)-Tg mice (Fig. 6D). Consistently, the western blot analysis showed that the expression of p16^INK4a^, p53 and p21 were markedly weaker in DLX3 (Q178R)-Tg mice compared with WT mice (Fig. 6E).

To examine if DLX3 mutation (Q178R) attenuates osteoblastic activity in a cell-autonomous manner, we isolated BMSCs from femurs of DLX3 (Q178R)-Tg and WT mice. Primary BMSCs from DLX3 (Q178R)-Tg mice demonstrated reduced osteogenic potential, as evidenced by ALP staining and Alizarin Red staining (ARS), when cells were induced by osteogenic medium (Fig. 6C).

## Discussion

Herein we demonstrate a distinct role for *DLX3* as an essential regulator of two integral processes in bone formation: osteogenesis and senescence. And our studies provide a possible explanation for abnormal bone formation of TDO patients and transgenic mice with *DLX3* mutation (Q178R). Owing to the inhibition of osteogenic potential found in BMSCs isolated from the TDO patient or transgenic mice, the phenotype of increased bone mineral density is attributed to delayed cellular senescence caused by *DLX3* mutation (Q178R). Additionally, the delayed skeletal aging and attenuated bone loss in *DLX3* mutation (Q178R)-Tg suggest that *DLX3* mutation is able to prevent aging-related bone loss. Mechanistically, the function studies of WT-DLX3, MT-DLX3 and TR-DLX3 in MC3T3-E1 extend our knowledge of DLX3 function in osteogenesis and senescence, as well as function alteration caused by DLX3 mutations.

Previous studies have proved that DLX3 is a key regulator of osteoblastogenesis. Yet, what’s the function of DLX3 in osteoblast differentiation? Most *in vitro* function studies have shown that *DLX3* have the capability to directly regulate osteogenic markers such as *OCN, RUNX2*, and osteoactivin^15^,^16^,^17^. Overexpression of DLX3 in osteoprogenitors promotes osteogenic differentiation, while knockdown of DLX3 suppresses^13^,^15^. However, these results are challenged by the experiments *in vivo*. Both of transgenic mice *Dlx3*^*Prx1-cKO*^ and *Dlx3*^*OCN-cKO*^ models, conditional loss-of-function mutations of *Dlx3* in mesenchymal cells and in osteogenic lineage cells respectively, present an increased bone mass accrual throughout the lifespan due to direct enhancement of osteoblast activity and up-regulation expression of osteogenic essential markers, including *Runx2* and its downstream target *Sp7*^14^. Undoubtedly, these results show that DLX3 is a negative regulator of bone mass accrual. Taken together, the studies above provide contrasting results and emphasize that DLX3 plays an implicated and sophisticated role in bone formation.

Given the fact that the bone homeostasis is an intricate process orchestrated by hundreds of factors, an interesting concept that emerges is that DLX3 performs its role in osteogenesis by coordinating with other osteogenic differentiation essential factors and constituting a regulatory network to keep the maintenance of bone homeostasis^36^. Firstly, *DLX3* gene belongs to a *Dlx* homeobox transcription factors family, which consists of three inverted and convergent clusters (*Dlx1/2, Dlx3/4* and *Dlx5/6*), and have essential function in skeleton development^2^,^37^,^38^. *Dlx5* and *Dlx6* deficient mice exhibits bone and ossification defects^2^,^38^. Increased levels of *Dlx5* and *Dlx6* are induced in transgenic mice with *Dlx3* deletion in osteogenic cells and are considered as a contributor to increased bone mineral density in the mice^14^. Hassan *et al*. show that the selection association of Dlx5, Msx2 and Dlx3 binding to the *Runx2* and *Ocn* promoter occurs in different stages of osteoblast maturation process^15^,^16^. Secondly, transcriptome studies exhibit that *in vivo Dlx3* inactivation in osteogenic cells affects a wide range of developmental signals including Wnt, TGF-β/BMP and Notch pathways^14^. Previous studies have already shown that BMP2 induces the osteogenic differentiation, and Dlx3 are rapidly induced in response to BMP2-mediated osteoblast differentiation. Hassan *et al*. prove a regulatory pathway from BMP2 to commitment to osteogenesis via Dlx3 regulation^16^. Furthermore, the mechanism of Dlx3 and BMP2 regulation each other with a feedback control is inspected in the osteogenic differentiation of dental follicle cells^39^. Of note, our study showed that BMP2 expression was lower in TDO-BMSCs and was decreased in MT-DLX3 but increased in TR-DLX3 (Supplementary Fig. S8), suggesting that these two different DLX3 mutations act as different proteins in triggering osteogenic differentiation regulatory network. Meanwhile, the different expression levels of BMP2 also explain the reason of inhibited osteogenic potential in MT-DLX3 and promoted in TR-DLX3. Thirdly, as the fatal member in bone homeostasis, osteoclast activity is a force to be reckoned with. The ration of receptor activator of NF-κB ligand (RANKL) and osteoprotegerin (OPG) is considered a marker of bone metabolism balance^40^. Isaac *et al*. demonstrate that Dlx3 deletion in osteoblasts gives rise to high levels of OPG, consequently resulting a decreased RANKL/OPG ratio and down-regulated bone resorption, and finally contributing to bone formation augment^14^. The phenotypes of increased bone volume and trabecular BMD in transgenic TDO mice (a mutant DLX3 (571_574delGGGG) driven by 2.3 Col1A1 promoter) are owing to decreased osteoclast formation and activity caused by increased IFN-γ, because the mice lack of enhanced dynamic bone formation *in vivo*^41^. Our study showed that DLX3 mutation (Q178R) in BMSCs leaded to a decreased RANKL/OPG ratio, which indicates a regulation balance against bone resorption. These results are further supported by our previous studies which reveal that DLX3 mutation (Q178R) inhibits osteoclast differentiation in Raw 264.7 cells by up-regulating osteoclastogenesis inhibitor microRNA-124 expression^42^. From this point, the two different DLX3 mutations share the same negative effect on osteoclasts activity.

Our functional studies in MC3T3-E1 showed that wild type DLX3 overexpression *in vitro* promoted osteogenic differentiation, which is consistent with the studies *in vitro* but opposite to the studies *in vivo*. Based on the concept of regulatory network to maintain bone homeostasis, it is possible that the *in vitro* studies exhibit DLX3 direct function due to the lack of regulatory network *in vitro*, whereas the *in vivo* studies demonstrate that other regulators concerning bone homeostasis in the network compensate or overcompensate the function alteration caused by DLX3 inactivation or mutations. This hypothesis is supported by the studies in the TDO transgenic mouse which demonstrated that the BMSCs isolated from these mice exhibit enhanced osteogenic potentials, but the dynamic bone formation is lacked in the mouse^41^. Of note, DLX3 expression in TDO-BMSCs was almost half of that in normal controls (Supplementary Fig. S4) and the TDO-BMSCs exhibited lower osteogenic potentials *in vitro*. Yet, increased bone density was observed in the TDO patient *in vivo*. These studies not only further testify the existence of regulatory network to keep bone homeostasis but also indicate that DLX3 mutation (Q178R) act as a loss of function *in vitro*. Furthermore, our DLX3 mutation (Q178R) transgenic mice studies reconfirm this concept by showing that BMSCs isolated from these mice exhibited decreased osteogenic potentials *in vitro*, but these mice demonstrated increased BMD and trabecular bone volume *in vivo*. Collectively, we are inclined to take DLX3 as a direct positive regulator in osteogenesis *in vitro*. As for the reason responsible for the conflicts, our studies offer the notion of senescence as the answer.

Our findings that delayed senescence in TDO-BMSCs remedied and enhanced the bone formation in TDO patient in spite of declined osteogenic potentials cast a novel insight of *DLX3* function in bone formation. In Isaac’s study, compared with the same age of wild type mice, loss of function of *Dlx3* in *Dlx3*^*OCN-cKO*^ mice results in femur BMD decreasing at 2 weeks to 5 weeks, but increasing from 4 months to the whole lifespan when compared with age matched wild-type mice^14^. These results indicate that *DLX3* plays an important role in bone formation when age is increased. Our immunohistochemical analysis demonstrated that osteoblasts senescence was delayed by *DLX3* (Q178R) mutation, and our Micro-CT analysis revealed that this delayed senescence attenuated aging-mediated bone loss *in vivo*. Overall, these experiments determine the role of *DLX3* in senescence. Mechanistically, the functional studies in MC3T3-E1 showed that wild type *DLX3* resulted in cell premature senescence, whereas both *DLX3* mutations prevented cellular senescence. These results imply that *DLX3* positively regulates cellular senescence and both *DLX3* mutations show a loss of function. In Palazzo’s study, the luciferase and EMSA assays demonstrates that Dlx3 regulated *p21* expression by directly binding to *p21* promoter or by co-regulating with p53^23^. Of note, DLX3 expression was lower in TDO-BMSCs (Supplementary Fig. S4). Taken together, DLX3 is therefore a positive regulator in senescence and it might regulate senescence through p53/p21-regulted network.

In general, evidence has accumulated that *DLX3* plays a pleiotropic role in several biological process to maintain bone homeostasis in normal bone, whereas, in TDO patients, this homeostasis is jeopardized by *DLX3* mutation through triggering different pathways in the regulatory network to rectify this mistake. In this TDO patient, *DLX3* (Q178R) mutation results in lower levels of expression of *RUNX2, OCN, ALP, BMP2, p16*^*INK4a*^, *p53, p21*, and so on, decreases osteogenesis and senescence. Mice lacking *p16*^*INK4a*^ demonstrate increased proliferation and reduced frequency of apoptotic events, as well as enhanced repopulation ability by raising expression of stem cell self-renewal genes^31^,^43^. *p21* deletion rescues proliferation and improves the repopulation capacity and self-renewal of stem cells from mice with dysfunctional telomeres^44^. The repressive expression of senescence genes rejuvenate BMSCs to keep the “young” state for their ability to self-renewal, proliferate, resistance to apoptosis and differentiate^24^,^45^,^46^, thereby more generations and longer lifespan of BMSCs compensate the decreased osteogenic differentiation propensity and finally lead to increased bone density.

Nevertheless, herein we focus on the role of *DLX3* mutation in senescence, even though it might not be the only reason for the increased bone formation. Its role highlights a concept that *DLX3* regulates bone formation by preventing senescence, gives us a novel sight to view *DLX3* function in bone formation. Furthermore, increasing bone-and age-related disorder, especially in the aging population, is becoming a major socioeconomic burden to society. The osteoporosis exhibits net bone loss on account of skeletal aging^47^. The application of BMSCs in bone repair and regeneration is limited because of its senescence causing a high rate of failure^48^. To tackle the challenge, novel insight into transcriptional programs bound up with bone formation and aging process are urgent to excavate novel therapeutic targets in future clinical trials. Our research of *DLX3* mutation protecting aging-related bone loss opens the possibility of its therapeutic potential in bone regeneration and bone loss disease.

## Methods

### Isolation and identification of BMSCs from the TDO patient and normal donors

A 26-year-old female with typical TDO features^11^ and gender- and age-matched normal donors (n = 3) participated in this study with informed consent. The study was approved by the Ethics Committee of Peking University School and Hospital of Stomatology. And the whole methods were carried out in accordance with the relevant guidelines, including any relevant details. BMSCs were isolated from alveolar bone of the involved patient (TDO-BMSCs) and healthy donors (CON-BMSCs) during implantation surgery as described below. Briefly, the bone tissue was rinsed in sterile phosphate-buffered saline (PBS), cut into 1 mm^3^ cubes, and digested in collagenase I (Invitrogen Life Technologies, Grand Island, NY, USA) and dispase II (Roche, Indianapolis, IN) for 1 h at 37 °C. Cells were collected by centrifugation at 200 × g and seeded in a 100-mm culture dish in alpha minimum essential medium (Alpha-MEM, Gibco, Paisley, Scotland, UK) supplemented with 10% fetal bovine serum (FBS) (Gibco) and penicillin-streptomycin and L-glutamine. Following overnight culture at 37 °C in a humidified atmosphere of 5% CO_2_ and 95% air, unattached cells were discarded when the medium was changed. Cells at passage 3 were analyzed for stem cell-associated markers by flow cytometry. Details were described in supplementary.

### Generation of transgenic mice and experimental animals

All animal experiments were approved by the Peking University Animal Care and Use Committee. All methods were performed in accordance with the relevant guidelines and regulations. Human *DLX3* mutation (Q178R) gene flanked by the mouse cytokeratin 14 promoter was subcloned into plasmid pGL647. The fragments of the DLX3 mutation (Q178R) transgene were purified and microinjected into C57BL/6 mouse oocytes, and the oocytes were surgically transferred to pseudopregnant C57BL/6 mouse (Cyagen Biosciences Inc., Guangzhou, China). Initial genotyping of founder transgenic mice was performed by PCR using tail biopsies from pups. Positive founders for transgene were reconfirmed by western blot analysis to test the overexpression of DLX3. Transgenic mice were bred with wild type mice to generate (to obtain a defined genetic background) hemizygotes for the analysis of bone phenotype changes. In all experiments, female transgenic mice and female wild type littermates as controls were used. We established a sample of at least 5 mice per group.

### Screening DLX3 status in BMSCs at DNA level

To screen DLX3 status in TDO- and CON-BMSCs, we collected cells from the patient and control donors at passage 1 and extracted DNA with the General AllGen Kit (Cwbiotech, Beijing, China) according to the manufacturer’s instruction. Exon 3 of DLX3 was amplified using GoTaq Green Master Mix (Promega, Madison, WI) and exon 3 primer pair. The forward exon 3 primer was 5′-AGCATTCTGAGAGGCTAACTAGCTAC-3′, and the reverse primer was 5′-AGGTTCTGTGCGTGATACCA-3′. The PCR products were then sequenced on ABI 3730 sequencer.

### Plasmids generation and cellular localization examination

To investigate the function of WT-DLX3 and MT-DLX3 protein, GFP-tagged DLX3 fusion gene expression plasmids were constructed. Briefly, Human full-length DLX3 cDNA was synthesized and subcloned into pEGFP-C1 vector. The novel mutant cDLX3 (c.533 A>G) and cDLX3 (c.571_574delGGGG) were generated from pEGFP-WT-DLX3 using single-site mutation kit (Promega, Madison, WI). The sequences of WT-DLX3, MT-DLX3 and TR-DLX3 were confirmed by DNA sequencing. To investigate the cellular localization, different plasmids were respectively transfected into MC3T3-E1 cells using Mirus TransIT-X2^®^ Dynamic Delivery System following the manufacturer’s instruction. At 48 h post-transfection, the cellular localization of WT-DLX3, MT-DLX3 and TR-DLX3 was examined using an inverted fluorescence microscope.

### Alkaline phosphatase (ALP) histochemistry and quantification of ALP activity

BMSCs were cultured in osteogenic medium for 3, 7, and 14 days. At the indicated times, the ALP histochemistry was stained with BCIP/NBT solution (Cwbiotech) and ALP activity was measured with an ALP assay kit (Nanjing Jiancheng Bioengineering Institute, Nanjing, China) according to the manufacturer’s instruction. The details were described in supplementary.

### Alizarin red staining and quantification of matrix mineralization

BMSCs were cultured in osteogenic medium for 14 and 21 days. At the indicated times, Alizarin red staining and quantification was performed. Details were described in supplementary.

### Measurement of β-galactosidase activity and expression in BMSCs

Senescence-associated β-galactosidase (SA-β-gal) activity was assessed using two methods. Firstly, the same number (5 × 10^5^) of BMSCs from both groups were seed into 60-mm culture dishes, and the medium was changed every 3 days. At 7 days post-inoculation, senescence was assessed using the senescence β-galactosidase staining kit (Beyotime Institute of Biotechnology, Jiangsu, China) according to the manufacturer’s instruction. To quantify SA-β-gal activity, we counted at least 300 cells in randomly choose fields and calculated the ratio of SA-β-gal^+^ cells to total cells. Secondly, to confirm the above result, BMSCs at passage 8 th were collected and analyzed by flow cytometry after incubation with anti-GLB1 antibody (Abcam, Cambridge, MA) and anti-human IgG isotype (GeneTex, Irvine, CA) for 1 h at 4 °C. The recommended PE-conjugated non-specific rabbit IgG [F(ab1)2 donkey anti-rabbit IgG PE secondary antibody (eBioscience, San Diego, CA)] was used as secondary antibody to measure GLB1 expression. Flow cytometry was performed on a Coulter Epics XL (Beckman Coulter, Inc.).

### Electrophoretic mobility shift assay (EMSA)

The nuclear extract (10 μg of protein) was mixed with biotin-labeled DNA probe or cold competitor in binding buffer (LightShift® Chemiluminescent EMSA Kit). The reaction mixture was incubated for 30 min at room temperature. GFP antibody was included for supershift. Then, the reaction mixture was incubated for additional 30 min at room temperature. Samples were run on an acrylamide gel at 120 V for about 2 h. The gel was transferred to a nylon membrane. The biotin-labeled DNA was detected using the streptavidin-horseradish peroxidase conjugate and LightShift chemiluminescent substrate.

### Telomere measurement by quantitative real-time PCR

Genome DNA was prepared using DNeasy Blood & Tissue Kit (Cwbiotech). Average telomere length was measured from total genomic DNA using a real-time PCR as previously described^29^,^30^. PCR reactions were performed on the ABI 7500 real-time PCR system (Life Technologies Corporation), using telomeric primers, primers for the reference control gene (human/mouse 36B4 single copy gene). For each PCR reaction, a standard curve was made by serial dilutions of known amounts of DNA. The telomere signal was normalized to the signal from the single copy gene to generate a T/S ratio indicative of relative telomere length. For human absolute telomere length calculation, the telomere kb per reaction value was divided genome copy number (calculated from 36B4 Ct and standard curve) to generate a total telomeric length in kb per diploid genome^49^. Equal amounts of DNA (20 ng) were used for each reaction, with at least three replicates for each specimen. The telomere standard curve and 36B4 standard curve was supplied in Supplementary Fig. S6.

### Real-time PCR analysis

Total RNA was isolated with TRIzol® reagent (Invitrogen Life Technologies) and 2 μg of RNA was reverse-transcribed into cDNA using the Superscript first-strand synthesis system (Invitrogen Life Technologies) according to protocol. Real-time PCR reactions were conducted in a 20-μl reaction mixture (containing cDNA and SYBR green master mix) using an ABI 7500 real-time PCR system (Life Technologies Corporation). The mRNA expression relative to osteoclastogenesis was normalized to murine glyceraldehydephosphate dehydrogenase (GAPDH) and calculated using the 2^−∆∆^ Ct method. The sequences of each primer are listed in Supplementary Tables S1 and S2.

### Western blot assay

Western blot assay was used to measure the protein expression, and the detailed procedures were described as below. Cells were harvested and lysed in RIPA buffer containing protease inhibitors. After determining the concentration, protein samples were subjected to 10% sodium dodecyl sulfate polyacrylamide gel electrophoresis and transferred to a polyvinylidenedifluoride membrane. The membranes were blocked in 5% skim milk for 1 h and incubated with antibodies against RUNX2, BSP, RANKL, OPG (Santa Cruz Biotechnology, Santa Cruz, CA) human p16^INK4a^, OCN, OCT4, EGFP (Cell Signaling Technology, Beverly, MA), p16^INK4a^, GLB1, DLX3 (Abcam), β-actin and GAPDH (Proteintech, Chicago, IL) separately at 4 °C overnight. After incubation with peroxidase-linked secondary antibodies, immunoreactive proteins were visualized on an Odyssey infrared imaging system (Odyssey LI-COR Biosciences, Lincoln, NE).

### Micro-CT analysis

Micro-CT analysis of specimens was performed using a high-resolution Inveon Micro-CT (Siemens, Munich, Germany). Briefly, the x-ray source was set at 60 kV, with a node current of 220 μA and an exposure time of 400 ms for each of the 360 rotational steps. Image slices were then reconstructed using micro-CT image analysis software (Inveon Research Workplace). The regions of interest were defined as the areas between 0.5 mm and 1.0 mm proximal to the growth plate in the distal femurs, in order to include the secondary trabecular spongiosa. A threshold of 500 was used for evaluation of all scans. For visualization, we imported the segmented data and reconstructed them as a 3D-image displayed in micro-CT image analysis software (Inveon Research Workplace).

### Immunohistochemical analysis

The specimens were decalcified in 10% ethylene diamine tetraacetic acid (EDTA) for 1 month, and sectioned (5 μm) for staining as previously described^50^. The specimens were incubated with primary antibody against p16^INK4a^ (Abcam) at 4 °C overnight. Then, samples were processed using the DAB detection kit (Zhangshanjinqiao, Beijing, China) and visualized under an Olympus BX51 light microscope equipped with an Olympus DP70 camera (Olympus, Co., Tokyo, Japan).

### Statistical analysis

All data was representative of each assay repeated independently at least three times with similar results. Statistical significance was determined using the two-tailed Student’s t-test, assuming equal variances. The χ^2^ test was used to compare rates. Significance is indicated as follows: *p < 0.05; **p < 0.01.

## Additional Information

**How to cite this article:** Zhao, N. *et al*. Senescence: novel insight into *DLX3* mutations leading to enhanced bone formation in Tricho-Dento-Osseous syndrome. *Sci. Rep.* **6**, 38680; doi: 10.1038/srep38680 (2016).

**Publisher's note:** Springer Nature remains neutral with regard to jurisdictional claims in published maps and institutional affiliations.

## Supplementary Material

Supplementary Information
