# Supplementary material for: Senescence: novel insight into DLX3 mutations leading to enhanced bone formation in Tricho-Dento-Osseous syndrome
Source: Sci Rep. 2016 Dec 7;6:38680. doi: 10.1038/srep38680 (PMC5141470; doi:10.1038/srep38680)
Supplement: Supplementary Information [file srep38680-s1.pdf]

Senescence: novel insight into *DLX3* mutations leading to enhanced bone formation in Tricho-Dento-Osseous syndrome

Na Zhao[1], Dong Han[1], Haochen Liu[1], Yue Li[1], Sing-Wai Wong[1,2], Zhengyi Cao[3], Jian Xu[4], Xiaowei Zhang[3], Tao Cai[5,6], Yixiang Wang[7*], Hailan Feng[1*]

[1]Department of Prosthodontics, Peking University School and Hospital of Stomatology

[2]Oral Biology Curriculum, School of Density, University of North Carolina at Chapel Hill

[3]Department of Biochemistry and Molecular Biology, Peking University Health Science Center

[4]School of Physics and Electronic Information, Henan Polytechnic University

[5]Institute of Genomic Medicine, Wenzhou Medical University

[6]National Institute of Dental and Craniofacial Research, NIH, Bethesda, MD

[7]Central Laboratory, Peking University School and Hospital of Stomatology

[*]Correspondence: Dr. Yixiang Wang, Central Laboratory, Peking University School and Hospital of Stomatology, 22 Zhongguancun Avenue South, Haidian District, Beijing 100081, China. Fax: 62173402, E-mail address: kqwangyx@bjmu.edu.cn; and Dr. Hailan Feng, Department of Prosthodontics, Peking University School and Hospital of Stomatology, 22 Zhongguancun Avenue South, Haidian District, Beijing 100081, China. Fax: 62173402, E-mail address: kqfenghl@bjmu.edu.cn. Phone number: 15652652734.

Conflicts of interest: The authors declare that they have no conflict of interest.

Senescence: novel insight into *DLX3* mutations leading to enhanced bone formation in Tricho-Dento-Osseous syndrome

Supplementary

Supplemental methods

Identification of BMSCs by flow cytometry analysis

Cells at passage 3 were analyzed for stem cell-associated markers by flow cytometry. After 3 washes with PBS-0.5% bovine serum albumin (BSA), the cells were incubated in the dark for 45 min at 4 ℃ with anti-human CD90-PE, CD105-PE, CD73-PE, CD166-PE, CD34-PE, and CD45-PE antibodies (BD Biosciences, San Jose, CA). The recommended non-specific human PE-conjugated IgGs (BD Biosciences) were used as an isotype control. Flow cytometry was performed on a Coulter Epics XL (Beckman Coulter, Inc., Brea, CA).

Alkaline phosphatase (ALP) histochemistry and quantification of ALP activity

The osteogenic medium was supplemented with 100 nM dexamethasone, 50 μg/mL L-ascorbic acid-2-phosphate, and 10 mM beta-glycerophosphate (Sigma-Aldrich, St. Louis, MO). BMSCs were cultured in such medium for 3, 7, and 14 days. The medium was changed every 3 days. At the indicated times, the cells were fixed in 95% ice-cold alcohol for 30 min, washed with Millipore-filtered water, and stained with BCIP/NBT solution according to the manufacturer's instructions. ALP activity was measured with an ALP assay kit according to the manufacturer's instruction. Concentrations of protein extracted from the cells were determined by the bicinchoninic acid protein assay (BCA reagent, Thermo Fisher Scientific Inc., Waltham, MA),

which was used to normalize the data.

Alizarin red staining and quantification of matrix mineralization

BMSCs were cultured in osteogenic medium supplemented with 100 nM dexamethasone, 50 μg/mL L-ascorbic acid-2-phosphate, and 10 mM beta-glycerophosphate. At day 14 and 21 after osteoinduction, calcium nodes were stained with 1% alizarin red S (Sigma-Aldrich). To quantify matrix mineralization, the stained samples were incubated in 100 mM cetylpyridinium chloride for 1 h to solubilize and release the calcium-bound Alizarin red into the solution. The absorbance of the released Alizarin red was measured at 562 nm.

Supplemental figure legend

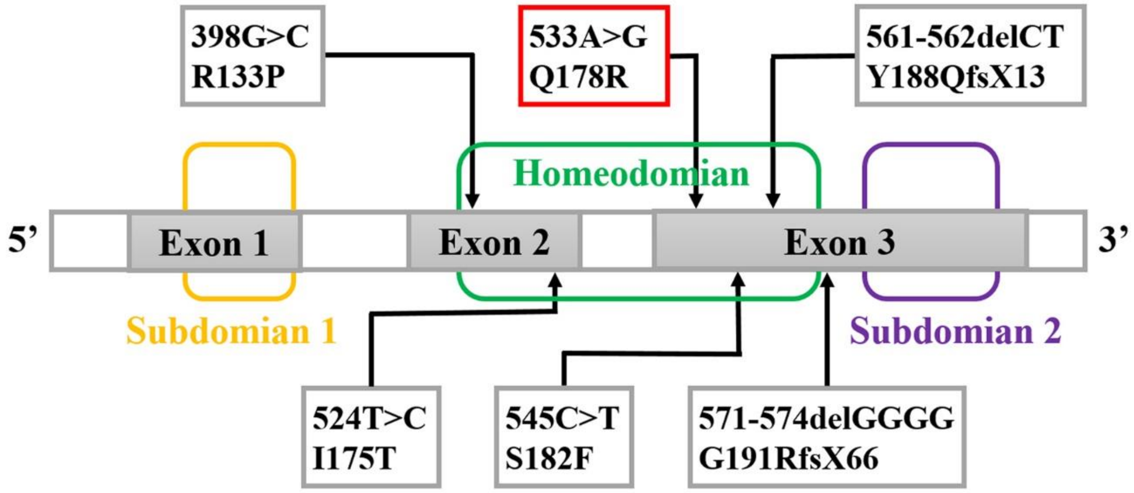

Supplemental Figure S1. Mutation analysis of the human *DLX3* gene.

The genomic structure of the *DLX3* gene includes three exons separated by two introns. The homeodomain is located in exons 2 and 3. Two subdomains are located on either side of the homeodomain in the DLX3 protein primary structure. A. c.571_574delGGGG (G191RfsX66),

reported in families from Virginia and North Carolina[1,2]. B. c.561_562delCT (Y188QfsX13), reported in a Korean family[3]. C.c.524T>C (I175T), a de novo missense mutation reported in an Austrian girl[4]. D. c.545C>T (S182F) and c.398G>C (R133P), reported in two Finnish families[5]. E. The c.533A>G (Q178R) mutation is a novel mutation reported in our previous study and framed in the red box[6].

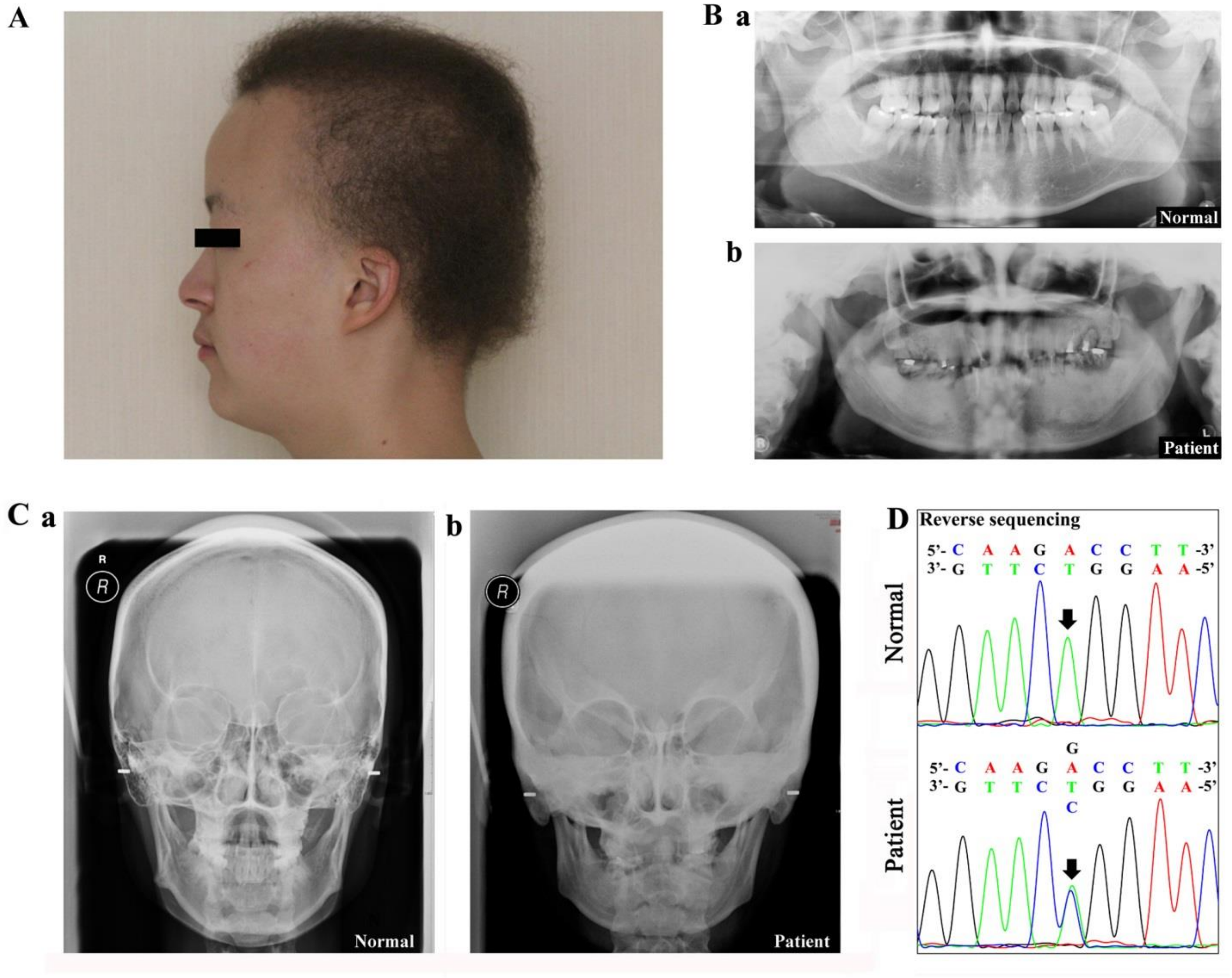

Supplemental Figure S2. Clinical characteristics of the TDO patient.

A. Lateral view: curly kinky hair and bossed frontal bone. B. Panoramic radiograph of normal people (a) and the TDO patient. Patient's panoramic radiograph showed clearly thickened

cortical and abnormally increased trabecular bone density of mandibular[7]. C. Cephalometric radiograph of normal people (a) and the TDO patient (b). Patient's cephalometric radiograph noted increased bone density and thickness in parietal and calveria bones, with absence of pneumatization of the mastoids. D. Reverse sequencing data of DLX3 exon 3 from the patient and a normal donor's BMSCs. Arrows indicated the mutation site.

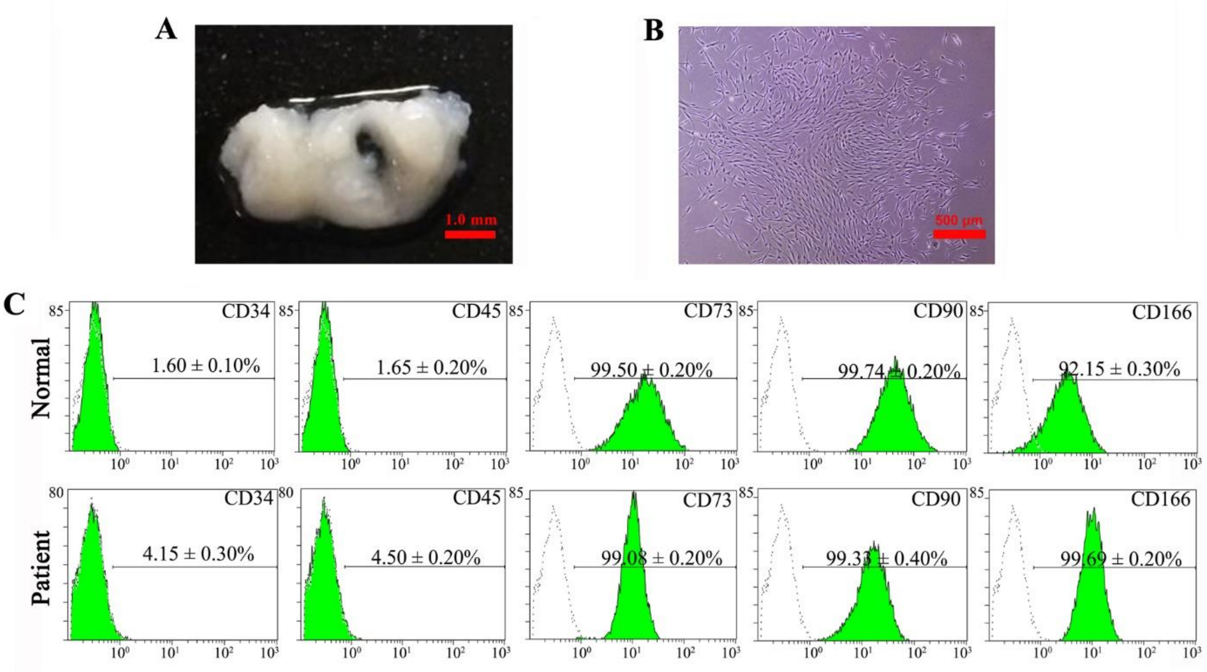

Supplemental Figure S3. Isolation, culture and characterization of human TDO-BMSCs.

A. Mandibular bone specimen from the TDO patient was used for isolation of TDO-BMSCs (scale bar, 1.0 mm). B. BMSCs from the TDO patient in primary culture reached 80% confluence after 14 days culture (scale bar, 500 μm). C. Flow cytometry data showing that TDO-BMSCs shared the same surface molecular profile as CON-BMSCs. Both BMSCs were positive for CD73, CD90, and CD166 and negative for CD34 and CD45. The dotted lines represented the staining of cells with corresponding isotype controls. Data were presented as the mean ± S.D. of 3 independent experiments.

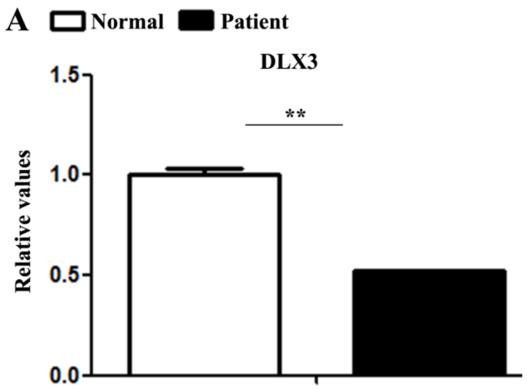

Supplemental Figure S4. *DLX3* expression in TDO patient's BMSCs.

A. Real time-PCR analysis of DLX3 expression in TDO patient's BMSCs compared with that of in normal controls. Data were presented as the mean ± S.D. of 3 independent experiments. **p <0.01.

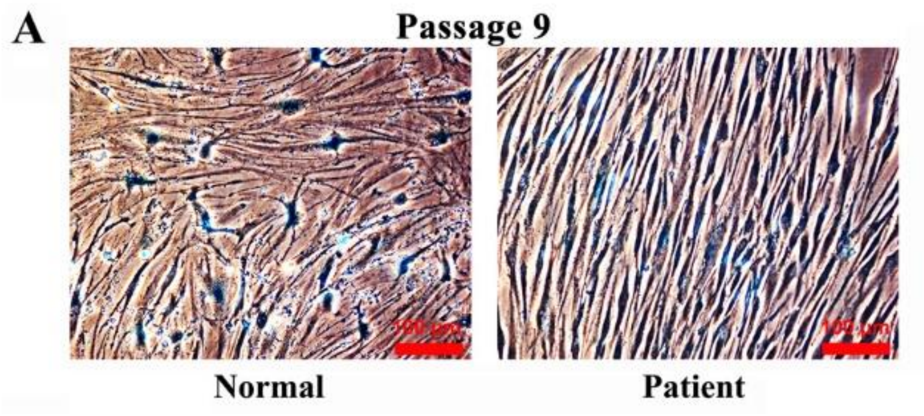

Supplemental Figure S5. Cell morphology difference between CON-BMSCs and TDO-BMSCs at passage 9.

Cell morphology were observed after 3 d culture with the same number cells seeded at passage 9. The classic senescence cell morphology, which was enlarged, flattened cellular morphology and

a decline replicate proliferation, was observed in CON-BMSCs. While TDO-BMSCs showed relative younger state, with regularly long spindle-shaped fibroblast-like morphology. On the other hand, the TDO-BMSCs number aggravated faster than CON-BMSCs with the same seed cells and the equal time, which further confirmed the results that TDO-BMSCs proliferated faster than CON-BMSCs at passage 9.

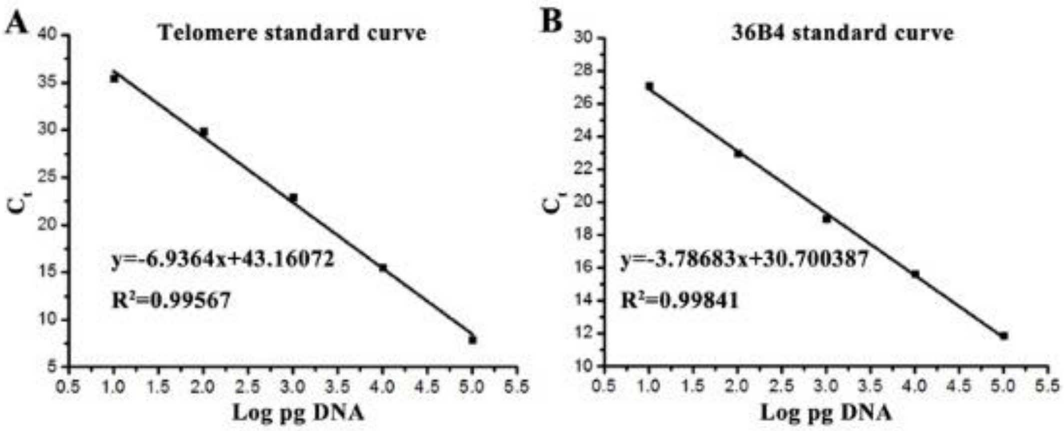

Supplemental Figure S6. Standard curves for telomere and single-copy gene 36B4 PCR.

Standard curves used to calculated relative DNA concentrations (log DNA) from C_t of the real-time PCR products by serial dilution of known amounts of DNA. C_t is the fractional number of PCR cycles at which a sufficient amount of the fluorescent product has been accumulated to cross a set magnitude threshold. Shown inside are the correlation regression equation and coefficients (R^2) of C_t versus log DNA.

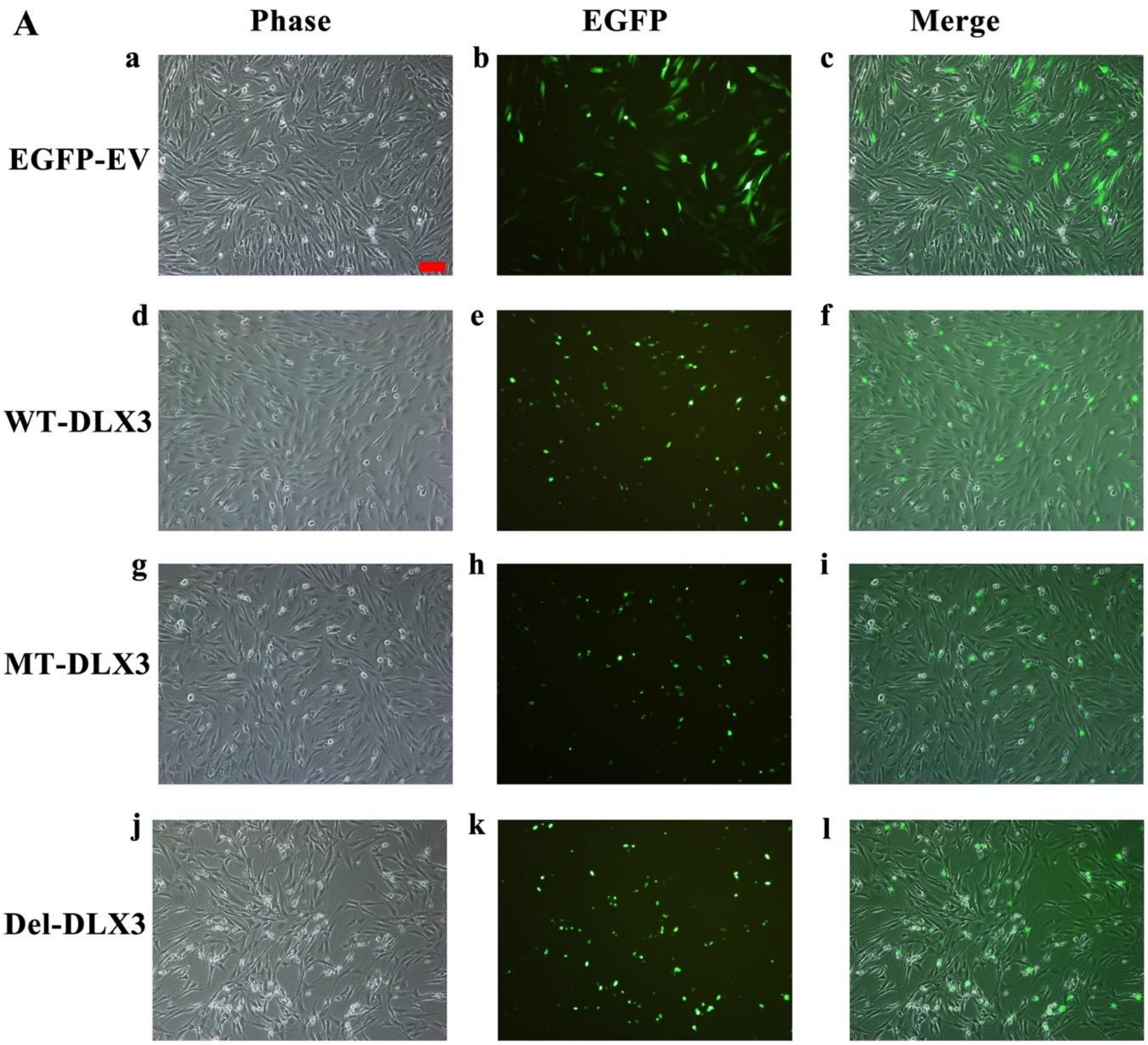

Supplemental Figure S7. Transfection efficiency of plasmids into MC3T3-E1.

A. Panels showing EGFP and EGFP-DLX3 fusion protein expression in MC3T3-E1 cells 48 h after transfection with pEGFP-C1, pWT-DLX3, pMT-DLX3 and pDel-DLX3. Differential interference contrast (DIC) images were acquired to visualize cell shape (a, d, g and j). Images of EGFP expression (b, e, h and k) were analyzed by fluorescent microscope. WT-DLX3, MT-DLX3 or Del-DLX3 was located in nuclear while the EGFP was in cytoplasm, which was shown in the merged images (c, f, i and l). The average transfection efficiency, calculated with at least 10 different scopes, was over 30%.

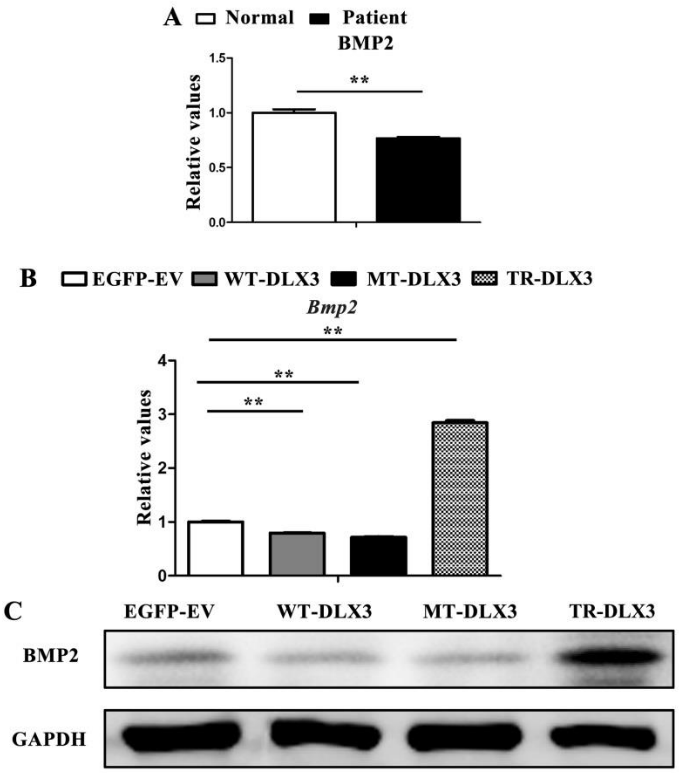

Supplemental Figure S8. BMP2 expression in TDO patient's BMSCs and MC3T3-E1 overexpression model.

A. Real time-PCR analysis of BMP2 expression in TDO patient's BMSCs compared with that of in normal controls. B&C: MC3T3-E1 cells were transfected with pEGFP-C1, pWT-DLX3, pMT-DLX3 and pTR-DLX3 respectively. After 24 h transfection, these cells were further cultured in osteoinduction medium for 72 h. Then, the cells were harvested and subjected to real-time PCR and western blot. B. *Bmp2* mRNA expression were determined by real-time PCR. GAPDH served as an internal control. C. Protein expression of BMP2 was detected by western blot. GAPDH served as loading control. Data were presented as the mean ± S.D. of 3 independent experiments. **p <0.01.

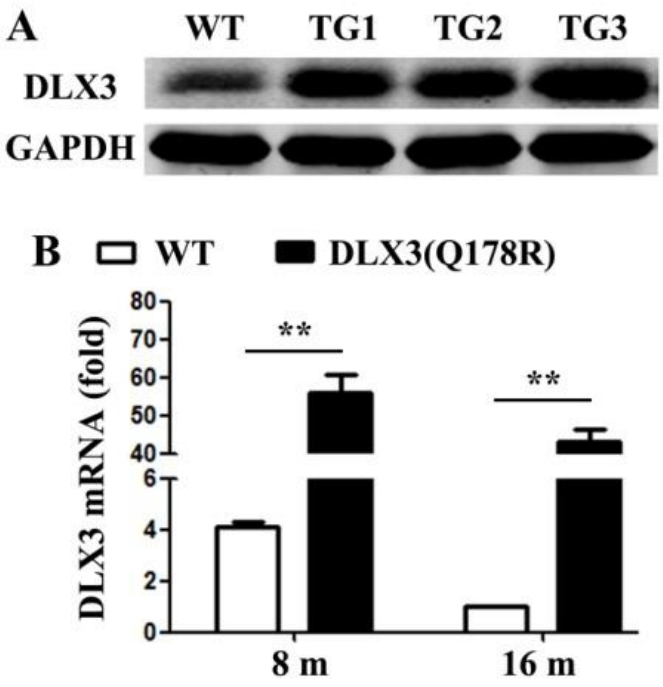

Supplemental Figure S9. DLX3 expression in mice.

A. Westernblot of DLX3(Q178R) transgene expression in 3 founder mouse lines. B. Real time-PCR analysis of DLX3 expression in in tibia and femur of 8- and 16- months old mice.

Supplemental Table S1. Real-time PCR primers of human used in this study

**Gene**	**Forward sequences**	**Reverse sequences**
GAPDH	CGACAGTCAGCCGCATCTT	CCAATACGACCAAATCCGTTG
RUNX2	AGGAATGCGCCCTAAATCACT	ACCCAGAAGGCACAGACAGAAG
ALP	GAACGTGGTCACCTCCATCCT	TCTCGTGGTCACAATGC
OCN	CCTGAAAGCCGATGTGGT	AGGGCAGCGAGGTAGTGA
BSP	CAGGCCACGATATTATCTTTACA	CTCCTCTTCTTCCTCCTCCTC
collagen I	CCCCTGGAAAGAATGGAGAT	AATCCTCGAGCACCCTGAG
RANKL	ACATATCGTTGGATCACAGCACAT	CAAAAGGCTGAGCTTCAAGCTT
OPG	GGAACCCCAGAGCGAAATACA	CCTGAAGAATGCCTCCTCACA

$p^{16INK4a}$	AGGAAGAAAGAGGAGGGGCT	TCATCATGACCTGGATCGGC
$p^{15INK4b}$	ACTAGTGGAGAAGGTGCGAC	GCCCATCATCATGACCTGGA
p53	GATGGAGAATATTTCACCCTTCA	CTGAGTCAGGCCCTTCTGTC
NANOG	CCAACATCCTGAACCTCAGC	GCTATTCTTCGGCCAGTTGT
OCT4	GTGGAGGAAGCTGACAACAA	CACTCGGTTCTCGATACTGG
DLX3	CTGGAGCACAGTCCCAATAAC	GCTGCTGCTGTAAGTGGGG

Supplemental Table S2. Real-time PCR primers of mouse used in this study

Gene	Forward sequences	Reverse sequences
Gapdh	AGGTCGGTGTGAACGGATTTG	TGTAGACCATGTAGTTGAGGTCA
Dlx3	ATTACAGCGCTCCTCAGCAT	CTTCCGGCTCCTCTTTCAC
Runx2	ATGCTTCATTCGCCTCACAAA	GCACTCACTGACTCGGTTGG
Alp	CCAACTCTTTTGTGCCAGAGA	GGCTACATTGGTGTTGAGCTTTT
Bsp	CAGGGAGGCAGTGACTCTTC	AGTGTGGAAAGTGTGGCGTT
$p^{16INK4a}$	ATCATCATCACCTGAATCGGGG	ATCATCATCACCTGAATCGGGG
$p^{15INK4b}$	TCTTGCATCTCCACCAGCTG	CTCCAGGTTTCCCATTTAGC
Oct4	GGCTTCAGACTTCGCCTCC	AACCTGAGGTCCACAGTATGC
Nanog	TCTTCCTGGTCCCCACAGTTT	GCAAGAATAGTTCTCGGGATGAA

References:

1. Price, J. A., Wright, J. T., Kula, K., Bowden, D. W. & Hart, T. C. A Common Dlx3 Gene Mutation is Responsible for Tricho-Dento-Osseous Syndrome in Virginia and North Carolina Families. *J Med Genet*. **35**, 825-828 (1998).

2. Price, J. A., Bowden, D. W., Wright, J. T., Pettenati, M. J. & Hart, T. C. Identification of a Mutation in Dlx3 Associated with Tricho-Dento-Osseous (Tdo) Syndrome. *Hum Mol Genet*. **7**, 563-569 (1998).

3. Lee, S. K. et al. Dlx3 Mutation in a New Family and its Phenotypic Variations. *J Dent Res*. **87**, 354-357 (2008).

4. Mayer, D. E., Baal, C., Litschauer-Poursadrollah, M., Hemmer, W. & Jarisch, R. Uncombable Hair and Atopic Dermatitis in a Case of Trichodento-Osseous Syndrome. *J Dtsch Dermatol Ges***. 8**, 102-104 (2010).

5. Nieminen, P. et al. Dlx3 Homeodomain Mutations Cause Tricho-Dento-Osseous Syndrome with Novel Phenotypes. *Cells Tissues Organs***. 194**, 49-59 (2011).

6. Li, Y. et al. Morphological Analyses and a Novel De Novo Dlx3 Mutation Associated with Tricho-Dento-Osseous Syndrome in a Chinese Family. *Eur J Oral Sci***. 123**, 228-234 (2015).

7. Li, Y. et al. Morphological Analyses and a Novel De Novo Dlx3 Mutation Associated with Tricho-Dento-Osseous Syndrome in a Chinese Family. *Eur J Oral Sci***. 123**, 228-234 (2015).
